# Knowledge, attitudes and practices related to neglected tropical diseases (schistosomiasis and fascioliasis) of public health importance: A cross-sectional study

**DOI:** 10.3389/fvets.2023.1088981

**Published:** 2023-02-28

**Authors:** Sajida Riaz, Haroon Ahmed, Sana Azeem Kiani, Muhammad Sohail Afzal, Sami Simsek, Figen Celik, Samia Wasif, Nazneen Bangash, Syed Kamran Naqvi, Jing Zhang, Jianping Cao

**Affiliations:** ^1^Department of Biosciences, COMSATS University Islamabad (CUI), Islamabad, Pakistan; ^2^Department of Life Sciences, School of Science, University of Management and Technology (UMT), Lahore, Pakistan; ^3^Department of Parasitology, Faculty of Veterinary Medicine, Firat University, Elazig, Türkiye; ^4^Department of Humanities, COMSATS University Islamabad (CUI), Islamabad, Pakistan; ^5^National Institute of Parasitic Diseases, Chinese Center for Disease Control and Prevention (Chinese Center for Tropical Diseases Research), Shanghai, China; ^6^Key Laboratory of Parasite and Vector Biology, National Health Commission of the People's Republic of China, Shanghai, China; ^7^WHO Collaborating Center for Tropical Diseases, Shanghai, China; ^8^The School of Global Health, Chinese Center for Tropical Diseases Research, Shanghai Jiao Tong University School of Medicine, Shanghai, China

**Keywords:** snail-borne parasitic diseases, survey, trematode, Pakistan, neglected tropical disease (NTD)

## Abstract

**Background:**

Snails play an important role as an intermediate host in various parasitic diseases, which are referred to as snail-borne parasitic diseases (SBPDs). The prevalence of the SBPDs, schistosomiasis and fascioliasis is low in Pakistan compared to other countries. The present study investigated knowledge, attitudes, and practices related to these two SPBDs and risk factors associated with them among the humans living in close contact with livestock and pets from three regions of Pakistan: Punjab, Islamabad and Azad Jammu and Kashmir (AJK).

**Methods:**

A cross-sectional survey was conducted using a structured questionnaire to assess participants' knowledge, attitude and practices related to schistosomiasis and fascioliasis during 2021–2022.

**Results:**

The majority of the 507 participants who were interviewed had good knowledge of schistosomiasis and fascioliasis: 43% were aware of schistosomiasis and 57% were aware of fascioliasis, but only 25% knew about snails as an intermediate host. Most respondents had a positive attitude toward treatment, prevention and control of the diseases but they did not have any healthcare facilities.

**Conclusion:**

This study highlights the importance of the public's awareness for the need to control SBPDs. It also draws attention to the need for health education for risk reduction and the prevention of SBPDs in endemic areas.

## 1. Introduction

Schistosomiasis, fascioliasis, clonorchiasis, fasciolopsiasis, paragonimiasis and opisthorchiasis are snail-borne parasitic diseases (SBPDs) that put humans' health at risk and are a major cause of the socio-economic losses of many countries. Snails act as intermediate hosts as well as transmitting vectors in SBPDs (1). The results of several studies have discussed ecological information on intermediate snail hosts and the parasites, but a very few have explained the fundamental role of snails in life cycle of snail-borne parasites (2). The snail is the intermediate host and the livestock is the final host in parasite's life cycle. Helminth parasites are the major causes of endemic production-limiting diseases of ruminant livestock worldwide (3). People with low helminth infections usually have no symptoms but heavy infections can cause a range of health problems. That includes abdominal pain, loss of blood and protein because worms feed on host blood and tissues, physical and cognitive growth obstruction (4). The most obvious and direct damage resulted from the pressure and blockage of internal organs exerted by growing parasites (5).

The prevalence of schistosomiasis and fascioliasis is low in Pakistan, compared to other countries. Schistosomiasis is a neglected tropical disease (NTD) caused by the genus *Schistosoma*, with a considerable impact on global health (6). Schistosomes are present in all geographical regions of the world, specifically in the developing countries of South America, Africa and Asia (WHO). Schistosomiasis, which has a water-based mode of transmission, *Schistosoma mansoni* and *S. haematobium* exists in endemic areas like Ethiopia (7). It is one of the 13 identified NTDs worldwide, although it is reported less often in Pakistan. In 1990, a survey of 20,000 cattle and buffaloes in northeastern Pakistan showed a 7–21% prevalence of schistosomes (8). In 2011, a survey of schistosomes on buffaloes conducted in the Punjab region showed prevalence rates of 13.6%−17% (9). Schistosomiasis in humans is not likely to be endemic in Pakistan. The 2001 reported case of a man who was diagnosed with schistosomiasis had actually acquired the infection from the country of its origin, Nigeria (10).

Fascioliasis, another NTD, the causative agent (*Fasciola* spp.) of the disease has two hosts life cycle; an intermediate host that is freshwater snail and the final hosts are livestocks, human and some other mammals. *Fasciola hepatica*, commonly known as liver fluke, which is a source of liver infection in sheep and cattle, is a zoonotic parasite that can transmit from animals to humans. Although onset of both diseases is low in the country as compared to other region of the world but still fascioliasis is widely spread throughout Pakistan unlike schistosomiasis which is quite less prevalent. In a 2012 study on the prevalence of fascioliasis in buffaloes in different agro-climatic areas in Pakistan, Bhutto et al. (11) randomly collected 1,800 fecal samples from buffaloes of different sexes and age groups, and found an overall fascioliasis prevalence of 42.06%. Another study reported the presence of human fascioliasis in different regions of Punjab (12), and a clinical assessment based study in Mardan (a district in the Khyber Pakhtunkhwa province of Pakistan) found only 4 (0.74%) children of 540 participants who tested positive for *Fasciola* eggs: two boys age 9- and 13-years and two girls age 7- and 16-years (13).

Higher rates of these diseases have emerged during the past decades, but have been neglected, making their prevention and treatment difficult, thereby posing a threat to the public health sector. Humans work in the following occupational categories: agriculture, animal husbandry, healthcare workers, slaughterhouse workers, farmers and housewives were with the highest risks for carrying fascioliasis and schistosomiasis. Earlier studies have shown an association between certain parasites and their intermediate host snails in general, but very few studies have focused on the central importance of snails and the mechanism and involvement of intermediate snail hosts in the complex life cycle of snail-borne parasites (2). Moreover, the basic biology of SBPDs and their hosts are very important to explain the geographical distributions of these diseases. Snail control is needed to avoid exposure to these diseases because their treatment is very difficult due to broad spectrum anthelmintic resistance (AR) in parasites of ruminants (14). Therefore, several practices should be used to control snails. This study was designed to examine the knowledge of humans living in close contact with livestock and pets (sometimes pet caught infection or eggs of parasites while visiting nearby contaminated sites) about the snail-borne diseases of schistosomiasis or fascioliasis and the role of snails in their transmission, attitudes toward schistosomiasis or fascioliasis and practices used to prevent these diseases, including snail control to prevent their transmission in Pakistan's Punjab, Islamabad, and Azad Jammu and Kashmir (AJK) regions.

## 2. Materials and methods

### 2.1. Study design

A quantitative cross-sectional study was conducted to assess participants' awareness and knowledge of schistosomiasis and fascioliasis. Data from the responses to a survey of people in the livestock industry, including those who slaughtered the animals, were analyzed. The average annual rate of slaughtered animals in Pakistan is 3.68%. As there are no recent or profound studies on this topic in Pakistan, the questionnaire was designed in accordance with the studies by Sady et al. (15) and Guan et al. (16) their study design was followed. The questionnaire, which was designed for the general population, was administered using face-to-face and online methods.

### 2.2. Study area and population

**Figure 1:**
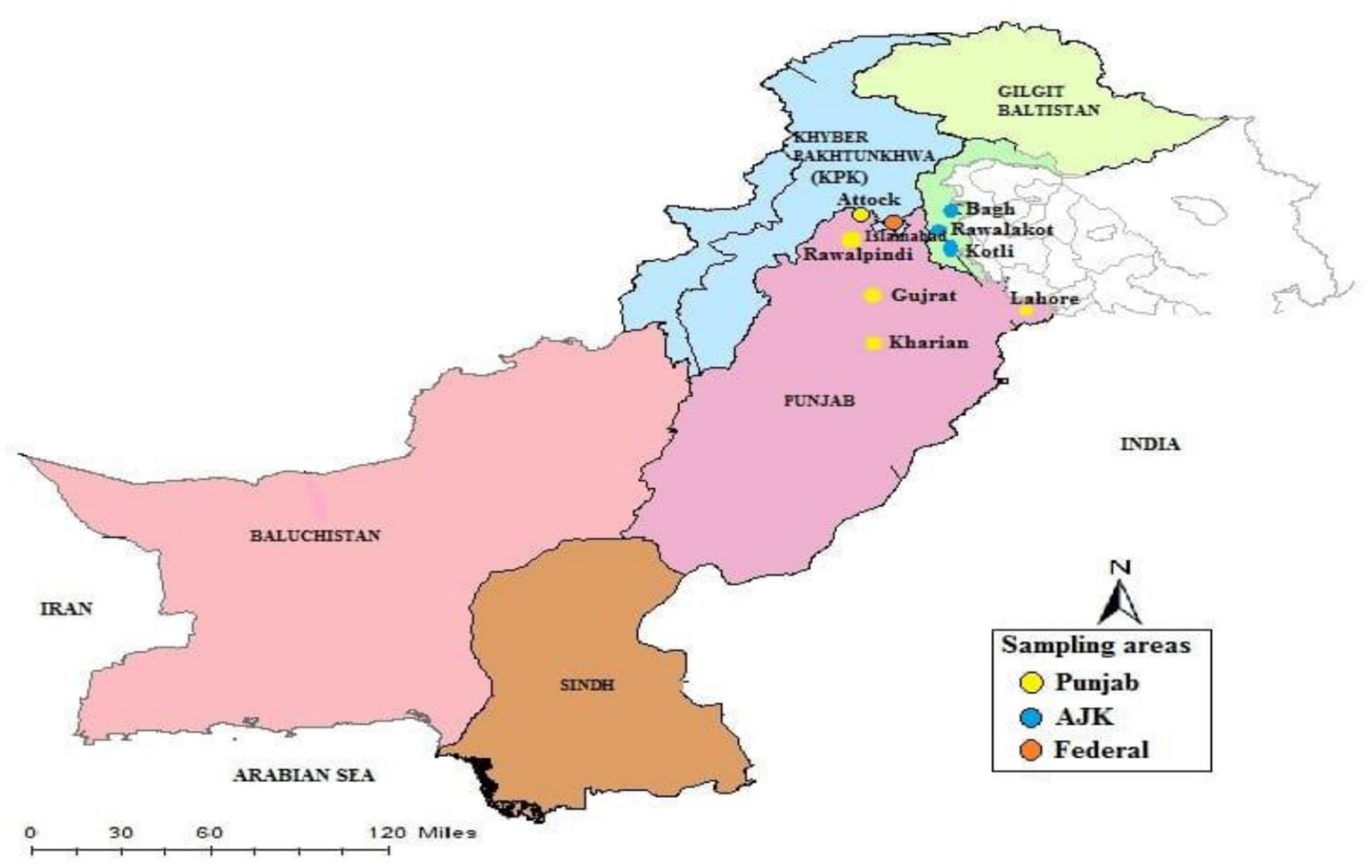
Map of the study area.

The study was conducted in rural and urban areas of Punjab (33.5651N and 73.0169E), Islamabad (33.6844N and73.0479E) and Azad Jammu and Kashmir (33.9259N and 73.7810E) as shown in Figure 1. Data were collected from a randomized population, which included both literate and illiterate people from different ethnic groups (i.e., Punjabi, Pakhtoon, Saraiki, and Urdu speaking). The study population from rural areas consisted of livestock owners, dairy farm workers and farmers who owned domesticated livestock, whereas the study population from urban areas was primarily composed of pet owners; both groups were living in close contact with their animals.

### 2.3. Study duration

This study's survey was conducted from August 2021 to February 2022 in the Punjab and Islamabad sampling regions of Pakistan and the AJK regions.

### 2.4. Data collection

A simple random sampling technique was used to collect the data. A questionnaire designed for the general population was filled online or in person, and face-to-face interviews were conducted. We personally circulate and filled the questionnaire at our own gadgets (Mobile and laptop etc.) and even translate it in local language while interviewing them.

### 2.5. Inclusion and exclusion criteria

People who had domestic animals, were 18 years of age or above and had knowledge relevant to this research were included in this study. The knowledge about the diseases, attitude and practices was added but the knowledge about prevalence was excluded. Children younger than 18 years of age, respondents whose questionnaires contained missing data or errors, or persons with any mental disability were excluded from the study.

### 2.6. Ethical approval

The Ethics Review Board of the Department of Biosciences, COMSATS University, Islamabad, approved the study (CUI/Bio/ERB/2021/44).

### 2.7. Sample size

The sample size was calculated using the Rao Soft Calculator (http://www.raosoft.com/samplesize.html), a 95% confidence interval (CI), 5% margin of error, a Z-score of 1.96 and a 50% response rate. Participants of different ages, genders, educational levels and locations were recruited to ensure the representativeness of the sample.

### 2.8. Study questionnaire

The questionnaire for the study was developed after a thorough review of the studies by Sady et al. (15) and Guan et al. (16). It was written in English but the interview questions were developed using the local languages of the participants (Punjabi and Urdu). A total of 98 questions were included in the questionnaire. Part 1 consisted of 10 questions to elicit information about participants' socio-demographic characteristics and 14 general information questions regarding livestock. A total of 25 questions in Part 2 assessed participants' knowledge, 27 questions in Part 3 assessed participants' attitudes, and 22 questions in Part 4 focused on participants' practices.

### 2.9. Data analysis

This study was based on an analysis of data collected in a survey of respondents from different areas of Pakistan and Azad Kashmir. The data collected were entered on an MS Excel spreadsheet, and the results were expressed as percentages and frequencies.

### 2.10. Statistical analysis for knowledge, attitude and practices

Statistical analyses were performed to investigate the significance of associations between the socio-demographic characteristics of respondents and their knowledge, attitude and practices. The associations between independent (socio-demographic) and dependent (knowledge, attitude and practices) variables were analyzed using binomial logistic regression analysis, with the significance level set to *P* < 0.05. Statistical analyses were performed using Jamovi Software, Version 2.2.2.

It was found that bivariate linear regression models could accurately predict participant's attitudes, knowledge as well as their own behaviors. First of all I had arranged all the responses from the study participants. After that, scoring was done with these responses i.e., response from every respondent of questions asked. Then average of responses was taken and grouping was done according to average. All the responses less than average value were considered as poor and rose responses above than average value were considered as good. Results of this statistical research indicate the elements that influence knowledge, attitudes and practices toward the fascioliasis and schistosomiasis. There was a statistically significant correlation between the odds ratios and the 95% confidence interval (CI) when the *P*-value was < 0.05.

## 3. Results

### 3.1. Socio-demographic characteristics

A total of 512 questionnaires were administered to participants in Punjab, Islamabad and AJK; 507 (99.0%) were included in the analysis and 5 were excluded from the analysis due to missing data and errors. Approximately 71.2% of the participants were females, 28.8% were males and more than 90.0% were married. The ethnicities of the participants were as follows: 138 (27.2%) were Punjabis, 3 (0.6%) were Sindhi, 22 (4.3%) were Pakhtoon, 152 (30%) were Kashmiris, 109 (21.5%) were Urdu speaking and 83 (16.4%) were from other ethnic groups. Approximately 10% of the participants were having bachelor's degree, 36.5% were intermediate, 49.5% were matriculated and 4% were below that. The majority of participants were livestock owners or handlers, 58.2% (295/507) were from rural areas and 41.8% (212/507) were belonging urban areas. We asked them how much time they spent with animals (hours per day) to assess their knowledge of livestock and livestock-associated diseases. Approximately 35.7% of participants spent fewer than 2 h or no time with their animals, 53.4% spent 3–5 h with them, 4.9% spent 6–10 h, and a few (2.6% and 3.4%) participants spent up to 15 h or more with their animals daily (Table 1).

**Table 1:** Participants' socio-demographic characteristics.

**Variable**	**Characteristic**	**Frequency (*n*)**	**Percentage (%)**
Gender	Male	146	28.8
	Female	361	71.2
Ethnicity	Punjabi	138	27.2
	Sindhi	3	0.6
	Pakhtoon	22	4.3
	Kashmiri	152	30
	Urdu speaking (Islamabad)	109	21.5
	Other	83	16.4
Religion	Muslim	506	99.8
	Non-Muslim	1	0.2
Marital status	Married	461	90.9
	Single	46	9.1
Education	No formal education	4	0.8
	Primary	2	0.4
	Middle	1	0.2
	Elementary	13	2.6
	Matriculation	251	49.5
	Intermediate	185	36.5
	Bachelors	48	9.4
	Masters	3	0.6
Residence	Rural	295	58.2
	Urban	212	41.8
No. of family members	< 5	118	23.3
	5–10	364	71.8
	11–15	19	3.7
	More than 15	6	1.2
Income/month (^*^PKRs)	Below 10,000/–	82	16.2
	10–20,000/–	61	12.0
	20–30,000/–	84	16.6
	Above 30,000/–	280	55.2
Time spent with animals (hours per day)	3–5	271	53.4
	6–10	25	4.9
	11–15	13	2.6
	More than 15	17	3.4
	Never or < 2 h	181	35.7

^*^Pakistani rupees.

### 3.2. Information about participants' livestock practices

Information was collected from participants about livestock and snails as vectors of helminth-borne diseases. Approximately 75.0% of participants knew that snails play a role in the life cycles of parasites, such as *Schistosoma* and *Fasciola*, but 25.0% were not aware of this. A total of 255 (50.3%) participants had a mixture of species (i.e., sheep, goats and cattle), 9.5% had sheep, 24.8% had goats, 14.2% had cattle and 1.2% had camels. Approximately 40.8% owned fewer than five animals and 3.2% owned more than 25 animals.; 27.2% raised single breeds and 23.8% raised mixed breeds; 164 (32.3%) participants had inbred livestock, 59 (11.7%) had outbred livestock and 56.0% of respondents did not know anything about breeding types. Among the participants, 62.7% were agro-pastoral farmers and 37.3% were pastoral farmers; 53.3% had an animal healthcare facility in their area while 20.3% did not have any nearby healthcare facilities for their animals. When asked if they had ever visited an animal clinic or took any of their animals to an animal clinic, 40.6% had taken an animal to a clinic, and the remaining 59.4% had never done so. The source of drinking water for 34.9% of the respondents was the farm, for 22.7% the source was a nearby stream and for 36.1% it was a nearby source of freshwater. In response to the question, “Have you ever found a snail on the land area where your animals graze or drink water?” 49.8% responded “yes,” 24.7% responded “no,” while the others responded that they did not know (Table 2).

**Table 2:** Information about livestock practices.

**Variable**	**Characteristic**	**Frequency (*n*)**	**Percentage (%)**
Do you know snails play a role in the life cycles of parasites, such as Schistosoma and Fasciola?	Yes	380	75.0
	No	127	25.0
How do you know?	Through formal education	175	34.5
	Through scientific knowledge	193	38.1
	Through friends	64	12.6
	Through news and social media	75	14.8
Livestock species (If you have)	Sheep	48	9.5
	Goats	126	24.8
	Cattle	72	14.2
	Camels	6	1.2
	Mixed species	255	50.3
Approximate No. of animals owned	< 5	207	40.8
	6–15	69	13.6
	16–25	12	2.4
	More than 25	16	3.2
	None	203	40.0
Type of herd raised	Single breed	138	27.2
	Mixed breed	121	23.8
	None	248	49.0
Type of livestock breeding	Inbreeding	164	32.3
	Outbreeding	59	11.7
	Not applicable	284	56.0
Animal slaughter method	Home slaughter	323	63.7
	Slaughter in an abattoir	184	36.3
Farming type	Agro-pastoral	318	62.7
	Pastoral	189	37.3
Presence of an animal healthcare facility in your area?	Yes	270	53.3
	No	103	20.3
	Do not know	134	26.4
Ever visit an animal clinic or taken any animal to an animal clinic?	Yes	206	40.6
	No	301	59.4
How do you treat a diseased animal?	Do not treat	99	19.5
	Use traditional methods	124	24.5
	Seek help from an animal healthcare provider	158	31.2
	Take the animal to a clinic	126	24.8
What is the type of land where your animal grazes?	Dry land	295	58.2
	Wet land	143	28.2
	Other	69	13.6
Where does your animal drink water?	On the farm	177	34.9
	At a nearby stream	115	22.7
	At a nearby freshwater source	183	36.1
	Other	32	6.3
Do you ever find snails in land areas where your animals graze or drink water?	Yes	252	49.8
	No	125	24.7
	Do not know	130	25.6

### 3.3. Participants' knowledge of helminth-borne diseases (schistosomiasis and fascioliasis)

Approximately 51.1 % of the participants were knowledgeable about zoonosis or zoonotic diseases; 60.7 % were aware of helminth-borne diseases; 57.0% were aware of fascioliasis; and 43% were familiar with schistosomiasis. More than 80% of the participants knew that fascioliasis or schistosomiasis could be diagnosed in animals, but the remaining 18% were unaware of this. An item that tested participants' knowledge of the names of diagnostic tests for fascioliasis or schistosomiasis showed that 53.2% knew the ELISA, 31 % knew the PCR and 15.8% knew both of these tests. Only 32.5% of participants had heard of a scheme, initiative or plan to control fascioliasis or schistosomiasis; 48.9% were familiar with infections in animals caused by snails; and 36.5% were aware of methods to control snails; but 63.5% were unaware of any snail-control methods (Table 3).

**Table 3:** Participants' knowledge of helminth-borne diseases (schistosomiasis and fascioliasis).

**Variable**	**Characteristic**	**Frequency (*n*)**	**Percentage (%)**
In what capacity do you have contact with animals?	Owner	288	56.8
	Herder	37	7.3
	Milker	79	15.6
	Dung cleaner	10	2.0
	All of above	93	18.3
Do you know that parasites can infect animals?	Yes	453	89.3
	No	54	10.7
Do you have any previous knowledge about zoonosis?	Yes	259	51.1
	No	248	48.9
Have you ever heard of helminth-borne diseases?	Yes	308	60.7
	No	199	39.3
If yes, with which helminth-borne diseases are you familiar?	Fascioliasis	289	57
	Schistosomiasis	218	43
How long have you known about schistosomiasis or fascioliasis?	1–5 years	222	43.7
	6–10 years	47	9.2
	11–15 years	26	5.1
	Not applicable	212	42
From which source did you acquire information about schistosomiasis or fascioliasis?	TV or radio	42	8.3
	Social media (FB/Twitter)	76	15
	From training session	23	4.5
	From awareness campaigns	49	9.7
	Veterinary staff	21	4.1
	Community health worker	12	2.4
	Relatives/family/friends	31	6.1
	Other	42	8.3
	Not applicable	211	41.6
Which symptoms are common in animals with schistosomiasis or fascioliasis?	General weakness	100	19.8
	Infertility	32	6.3
	Reduced milk production	38	7.5
	All of above	128	25.3
	Do not know	209	41.2
Can schistosomiasis or fascioliasis be transmitted from animals to humans?	Yes	372	73.4
	No	135	26.6
How is schistosomiasis or fascioliasis transmitted?	Through contact with infected animal	153	30.2
	Consuming infected dairy products	73	14.4
	Both	199	39.2
	None	82	16.2
What are the important risk factors for schistosomiasis or fascioliasis in animals?	Climatic conditions	90	17.7
	Species	52	10.3
	Herd size	38	7.5
	Geography	27	5.3
	Age and sex	29	5.8
	Residence conditions and feeding	164	32.3
	None of above	107	21.1
How can schistosomiasis or fascioliasis be prevented in animals?	Proper vaccination	102	20.1
	Isolating infected animals	52	10.3
	Minimizing risk factors	70	13.8
	All of above	170	33.5
	Don't know	113	22.3
Can schistosomiasis or fascioliasis be diagnosed in animals?	Yes	412	81.3
	No	95	18.7
Which diagnostic test used for fascioliasis and schistosomiasis have you heard about?	ELISA	270	53.2
	PCR	157	31
	Both	80	15.8
How do you identify schistosomiasis or fascioliasis in an infected animal?	By decreased milk production	73	14.4
	By general weakness	71	14.0
	By loss of appetite	56	11.0
	By the large size of her abdomen	34	6.7
	All of above	170	33.5
	None of above	103	20.3
Have you seen any schistosomiasis or fascioliasis infected animals in your area?	Yes	334	65.9
	No	173	34.1
Do you know about the health related threats posed by contaminated dairy products?	Yes	296	58.4
	No	211	41.6
Do you know that schistosomiasis or fascioliasis can be transmitted through blood transfusions?	Yes	256	50.5
	No	251	49.5
Do you know that schistosomiasis can be transmitted to humans when their cercaria penetrate the skin?	Yes	247	48.7
	No	260	51.3
Have you ever heard of any schemes, initiatives or plans to control schistosomiasis or fascioliasis?	Yes	165	32.5
	No	342	67.5
Have you ever heard of infections in animals caused by snails?	Yes	248	48.9
	No	259	51.1
Have you ever heard of any methods for snail control?	Yes	185	36.5
	No	322	63.5

### 3.4. Participants' attitudes toward schistosomiasis and fascioliasis

There are people who consider the two diseases a serious problem for both animals and humans. Approximately 34.9% (177/507) of the respondents considered schistosomiasis and fascioliasis serious animal diseases and 9.5% (48/507) considered them serious human ailments. Most (65.5%) participants vaccinated their animals but 34.5% did not provide any vaccinations for their animals; 34.1% had attended a training, awareness session or workshop related to livestock diseases; and 71.2% supported initiatives taken to control schistosomiasis or fascioliasis. Approximately 63.7% of respondents said they would visit a doctor and 14.2% would self-medicate if a human at a livestock facility had symptoms associated with either of these diseases. To prevent onset of diseases 48.5% of respondents reported that they would seek vaccination, 17.0% would contact a vet, 16.2% would isolate the infected animals and 18.3% had no proper response. In response to the item about how schistosomiasis or fascioliasis infection can be cured, 55.2% of the respondents reported they would visit any healthcare facility (Table 4).

**Table 4:** Attitudes of participants toward schistosomiasis and fascioliasis.

**Variable**	**Characteristic**	**Frequency (n)**	**Percentage (%)**
Are you informed when an animal is sick?	Yes	324	63.9
	No	183	36.1
What is your perception of schistosomiasis or fascioliasis?	Serious animal disease	177	34.9
	Serious human disease	48	9.5
	Both	175	34.5
	None	107	21.1
Have you attended any training, awareness session or workshop related to livestock diseases?	Yes	173	34.1
	No	334	65.9
Will you support any initiative taken to control helminth borne diseases (schistosomiasis or fascioliasis)?	Yes	361	71.2
	No	146	28.8
Will you support any initiative taken to control snails that host *Schistosoma* and *Fasciola* parasites?	Yes	353	69.6
	No	154	30.4
Do you think that diseases, such as schistosomiasis and fascioliasis affect livestock production?	Yes	377	74.4
	No	130	25.6
Which measures have you taken or would take to treat an animal infected with schistosomiasis or fascioliasis?	Seek professional help	233	46.0
	Vaccinate	126	24.9
	Isolate the infected animal	60	11.8
	Treat using traditional methods	29	5.7
	Will do nothing	59	11.6
To whom do you sell your animals most regularly?	Local market	207	40.8
	Slaughterhouse	100	19.7
	General community	200	38.5
Attitude toward infected animals	Sell the animal	60	12.0
	Inform a veterinary officer	175	34.5
	Isolate the animal	125	24.6
	Slaughter the animal	56	11.0
	None	91	17.9
Do you know what happened to the animals you sold e.g., were they slaughtered?	Yes	249	49.1
	No	258	50.9
After an animal is slaughtered, how are the infected parts sorted?	Discarded	264	52.0
	Sold after removing infected part	147	29.0
	Sold as it is	96	19.0
Are Fasciola-infected livers ever sold to buyers?	Yes	217	42.8
	No	290	57.2
Is the dung of slaughtered animals removed?	Yes	360	71.0
	No	147	29.0
What happens to the intestines of slaughtered animals? Are they	Cleaned	225	44.4
	Disposed of	282	55.6
If cleaned, then with	Bare hands	158	31.2
	Gloved hands	349	68.8
If discarded, then they are	Disposed of underground	344	68.0
	Disposed of open	163	32.0
Is the disposal site	Aquatic	147	29.0
	Terrestrial	360	71.0
Do humans and other animals have contact at the disposal site?	Yes	257	50.7
	No	250	49.3
Do other stray animals graze or drink at the disposal sites?	Yes	252	49.7
	No	255	50.3
Have you known about any cases of abortion in animals infected with schistosomiasis or fascioliasis?	Yes	162	31.9
	No	345	68.0
How the health of animal is ensured when you/the owner are buying it or when you are receiving new cattle?	Seek veterinary advice	210	41.4
	Rely on own experience	85	16.8
	Buy from known and/or trusted people	117	23.1
	None	95	18.7
What would you do if a person at your livestock facility had symptoms generally associated with schistosomiasis or fascioliasis?	Go to a doctor	323	63.7
	Self-medicate	72	14.2
	Go to a traditional healer	27	5.3
	None	85	16.8
	None	84	16.6
Schistosomiasis or fascioliasis can be prevented in animals by	Vaccination	246	48.5
	Contacting a veterinary office	86	17.0
	Isolation of infected animals	82	16.2
	Not applicable	93	18.3

### 3.5. Participants' practices for the prevention of schistosomiasis and fascioliasis

Only 30.0% of the 507 participants attended training to handle livestock; the remaining 70.0% handled their animals without any proper training, but if any of their animals were sick, 71.8% reported that they separated the sick from the healthy animals, whereas the remaining 28.2% did not practice isolation. Approximately 66.5% of participants reported using gloves while disposing fecal material or other discharges, whereas 33.5% did not use gloves. Only 47.2% of respondents quarantined newly purchased animals for a specific duration, while 52.8% did not take such precautions. Approximately 79.1% of participants washed their hands before and after milking animals and while consuming milk; 80.5% participants boiled raw milk but 65.1% also used raw milk to make other dairy products, such as lassi, butter, and ghee. Approximately 54.0% of respondents mixed their livestock with other animals, 28.6% separated their animals by species, 9.7% separated them by age and 7.7% did so by sex. Approximately 62.1% used protective clothing while handling animals, and the remaining 37.9% did not use any protective clothing. About 48.0% who owned livestock, lent their male animals to other herds.

Approximately 68.6% of the participants sent their animals to common grazing areas, 31.4% fed their animals at their own place, and 37.5% shared their living space with the animals. To maintain hygiene in the animal areas, 61.5% disinfected the spaces where their animals were kept, 38.5% did not disinfect the areas, and 36.7% removed dung from the livestock facility daily. Approximately 26.2% kept their dung piles to be used as fertilizer for 1–3 months, 14.2% kept them for 3–6 months, 7.1% for more than 6 months and 52.5% did not store dung. The animals of 35.5% of the participants had access to dung areas; 66.1% of the participants cleaned all of the feeding and water troughs, but only 41.8% practiced methods to eliminate snails from their animals' feeding and water troughs, which are a major source of *fasciola* and *schistosoma* cerceria transmission. Approximately 49.3% of participants slaughtered their animals in their livestock facilities while 50.7% slaughtered their animals in butcher shops, slaughterhouses or farms (Table 5).

**Table 5:** Participants' practices for preventing the spread of schistosomiasis and fascioliasis.

**Practices**	**Characteristic**	**Frequency (*n*)**	**Percentage (%)**
Have you attended any particular training for handling livestock?	Yes	152	30.0
	No	355	70.0
Do you separate sick animals from healthy animals?	Yes	364	71.8
	No	143	28.2
Do you use gloves while disposing fecal material or other discharges?	Yes	337	66.5
	No	170	33.5
Do you keep newly purchased animals in quarantine for some time?	Yes	239	47.2
	No	268	52.8
Do you boil milk before consumption?	Yes	408	80.5
	No	99	19.5
How do you keep your livestock animals?	Mixed	274	54.0
	Species separated	145	28.6
	Age separated	49	9.7
	Sex separated	39	7.7
Do you use any type of protective clothing while handling animals?	Yes	315	62.1
	No	192	37.9
Do you lend the male animals of your herd to other herds?	Yes	243	48.0
	No	264	52.0
Do you use raw milk to make other dairy products (lassi, butter, ghee etc.)?	Yes	330	65.1
	No	177	34.9
Do you send your animals to common grazing areas?	Yes	348	68.6
	No	159	31.4
Do you disinfect the space where your animals are kept?	Yes	312	61.5
	No	195	38.5
Do you wash your hands before and after milking?	Yes	401	79.1
	No	106	20.9
Do you live in shared places with animals?	Yes	190	37.5
	No	317	62.5
What is the dung cleaning routine in your livestock facility?	Clean dung daily	186	36.7
	Regularly	142	28.0
	Once a week	56	11.0
	Occasionally	30	6.0
	Never	93	18.3
How long (in months) do you store dung piles?	1–3	133	26.2
	3–6	72	14.2
	>6	36	7.1
	Don't store	266	52.5
Do other animals have access to stored dung piles?	Yes	180	35.5
	No	327	64.5
Is calving space shared with other animals?	Yes	199	39.3
	No	308	60.7
Do you slaughter animals at your livestock facility?	Yes	250	49.3
	No	257	50.7
Do you clean the feeding and water troughs?	Yes	335	66.1
	No	172	33.9
Do you practice any method to eliminate snails from your animals' feeding and water troughs?	Yes	212	41.8
	No	295	58.2
Have you ever been subjected to a blood donation?	Yes	174	34.3
	No	333	65.7
Have you ever received blood?	Yes	124	24.5
	No	383	75.5

### 3.6. Associations between participants' knowledge of helminth-borne diseases and socio-demographic characteristics

For binomial logistic regression we find association between the independent (socio-demography) and the dependent (knowledge, attitude and practices) variables considering *P* values ≤0.05 as significant was. One of each independent variable was used as a reference category in the analysis of the associations. Significant associations between participants with intermediate and bachelor's levels of education and knowledge about schistosomiasis or fascioliasis were found (Table 6). This table describe the knowledge of the participants with association of the sociodemographic characteristics. Partipants having the intermediate level of education and bachelor have the significant *P* values 0.019 and 0.017, respectively are predictors of less knowledge as compared to their reference level that was matriculation level. Significant *P* value (<0.05) indicates the greater deviation between the observed values of respondents in these education levels and reference value. This significant difference occurs because we are comparing all the groups from responses of different questions by respondents with reference level.

**Table 6:** Associations between participants' knowledge and socio-demographic characteristics.

**Variable**	**Characteristic**	**Knowledge status**		**Estimate**	SE^*^	**Z-score**	***P*-value**	Odds ratio (95% ^*^CI)	**R^2^_McF_**
		**Good**	**Poor**						
Gender	Male	109	37	−0.107	0.224	−0.479	0.632	0.898 (0.579–1.393)	3.92e-4
	**Female**	**262**	**99**						
Marital status	Married	333	128	0.602	0.403	1.50	0.135	1.826 (0.8293–4.020)	0.00422
	Single	38	8						
Education	No formal education	1	3	1.8341	1.163	1.5776	0.115	6.259 (0.641–61.110)	0.0315
	Primary	2	0	−14.8306	1029.121	−0.0144	0.989	3.62e−7 (0.000–inf)	
	Middle	1	0	−14.8306	1455.398	−0.0102	0.992	3.62e−7 (0.000–inf)	
	Elementary	9	4	−0.0755	0.616	−0.1225	0.902	0.927 (0.277–3.101)	
	Matriculation	169	81						
	Intermediate	144	41	−0.5208	0.223	−2.3385	^*^0.019	0.594 (0.384–0.919)
	Bachelors	41	7	−1.0322	0.431	−2.3965	^*^0.017	0.356 (0.153–0.829)
	Masters	4	0	−14.8306	727.699	−0.0204	0.984	3.62e−7 (0.000–inf)
Residential area	Rural	208	87	0.324	0.207	1.57	0.118	1.383 (0.922–2.075)	0.00422
	Urban	162	49						
No. of family members	5–10	266	98	0.03109	0.534	0.05820	0.954	1.032 (0.362–2.939)	2.48e−4
	< 5	87	31	−0.00230	0.561	−0.00410	0.997	0.998 (0.332–2.998)	
	More than 15	4	2	0.33647	1.011	0.33292	0.739	1.400 (0.193–10.148)	
	11–15	14	5						
Income per month (^*^PKRs)	Below 10,000	57	25	0.690	0.410	1.68	0.093	1.994 (0.892–4.457)	0.00681
	11,000–20,000	50	11						
	21,000–30,000	57	26	0.729	0.409	1.78	0.074	2.073 (0.931–4.618)	
	Above 30,000	207	74	0.485	0.360	1.35	0.177	1.625 (0.803–3.287)	

* P < 0.05; SE, standard error; CI, confidence interval; PKRs, Pakistani rupees.

### 3.7. Associations between participants' attitude and socio-demographic characteristics

No significant association between participants' socio-demographic characteristics and attitude toward snail-borne parasitic diseases i.e., fascioliasis and schistosomiasis were found in this study (Table 7).

**Table 7:** Associations between participants' attitude and socio-demographic characteristics.

**Variable**	**Characteristic**	**Attitude**		**Estimate**	^*^SE	**Z-score**	***P*-value**	Odds ratio (95% ^*^CI)	**R^2^_McF_**
		**Positive**	**Negative**						
Gender	Male	128	18	−0.0131	0.299	−0.0437	0.965	0.987 (0.550–1.77)	5.06e−6
	Female	317	44						
Religion	Muslim	444	61						6.91e-4
	Non-Muslim	1	0	12.58	882.743	0.0143	0.989	291011.21 (0.00–inf)	
Marital status	Married	404	57	−0.146	0.494	−0.295	0.768	0.864 (0.328–2.28)	2.38e−4
	Single	41	5						
Education	No formal education	2	2	−1.782	1.016	1.75422	0.079	0.168 (0.0230–1.23)	0.0301
	Primary	2	0	14.784	1696.734	0.00871	0.993	2.63e+6 (0.0000–inf)	
	Middle	1	0	14.784	2399.545	0.00616	0.995	2.63e+6 (0.0000–inf)	
	Elementary	13	0	14.784	665.514	0.02221	0.982	2.63e+6 (0.0000–inf)	
	Matriculation	214	36						
	Intermediate	165	20	0.328	0.298	1.10167	0.271	1.388 (0.7746–2.49)	
	Bachelors	45	3	0.926	0.623	1.48594	0.137	2.523 (0.7443–8.55)	
	Masters	3	1	−0.684	1.169	−0.58515	0.558	0.505 (0.0511–4.99)	
Residential area	Rural	262	33	0.195	0.274	0.709	0.478	1.21 (0.709–2.08)	0.00134
	Urban	183	28						
No. of family members	5–10	321	43	0.688	0.586	1.1755	0.240	1.99 (0.632–6.27)	0.00748
	< 5	103	15	0.605	0.627	0.9649	0.335	1.83 (0.536–6.26)	
	More than 15	6	0	14.244	594.164	0.0240	0.981	1.54e+6 (0.000–inf)	
	11–15	15	4						
Income per month (^*^PKRs)	Below 10,000	72	10	0.220	0.494	0.445	0.656	1.25 (0.473–3.28)	0.00108
	11,000–20,000	52	9						
	21,000–30,000	73	10	0.234	0.494	0.473	0.636	1.26 (0.480–3.33)	
	Above 30,000	248	33	0.263	0.406	0.648	0.517	1.30 (0.587–2.88)	

* SE, standard error; CI, confidence interval; PKRs, Pakistani rupees.

### 3.8. Associations between participants' practices and socio-demographic characteristics

The associations between participants' practices for controlling snail-borne parasitic diseases (i.e., schistosomiasis and fascioliasis) are shown in Table 8. Most of these associations were non-significant, the predictors of less practice were respondents of rural areas (Odds ratio 1.530, *P*-value 0.047) than their reference level that was respondents of urban areas. This was because of the reason that people of rural areas are not habituated to hygiene as people of urban areas, so lack basic hygiene practices for preventing schistosomiasis and fascioliasis.

**Table 8:** Associations between participants' practices and socio-demographic characteristics.

**Variable**	**Characteristic**	**Practice**		**Estimate**	^*^SE	**Z-score**	***P*-value**	Odds ratio (95% ^*^CI)	**R^2^_McF_**
		**Good**	**Poor**						
Gender	Male	109	37	0.0219	0.226	0.0969	0.923	1.022 (0.657–1.591)	1.64e−5
	Female	271	90						
Religion	Muslim	379	126						0.00101
	Non-Muslim	1	0	−12.46	535.411	−0.0233	0.981	3.86e−6 (0.000–inf)	
Marital status	Married	344	117	0.202	0.373	0.543	0.587	1.224 (0.589–2.544)	5.32e−4
	Single	36	10						
Education	No formal education	1	3	2.023	1.163	1.7395	0.082	7.563 (0.774–73.930)	0.0198
	Primary	2	0	−13.641	624.194	−0.0219	0.983	1.19e−6 (0.000–inf)	
	Middle	1	0	−13.641	882.743	−0.0155	0.988	1.19e−6 (0.000–inf)
	Elementary	10	3	−0.279	0.673	−0.4149	0.678	0.756 (0.202–2.829)
	Matriculation	179	71					
	Intermediate	147	38	−0.428	0.230	−1.8634	0.062	0.652 (0.415–1.022)
	Bachelors	38	10	−0.410	0.382	−1.0738	0.283	0.663 (0.314–1.403)
	Masters	2	2	0.925	1.010	0.9157	0.360	2.521 (0.348–18.244)
Residential area	Rural	212	83	0.425	0.214	1.98	^*^0.047	1.530 (1.005–2.329)	0.00707
	Urban	168	43						
No. of family members	5–10	275	89	0.194	0.576	0.336	0.737	1.214 (0.3926–3.752)	0.00124
	< 5	86	32	0.333	0.600	0.556	0.578	1.395 (0.4308–4.519)	
	More than 15	4	2	0.629	1.033	0.609	0.543	1.875 (0.2477–14.194)	
	11–15	15	4						
Income per month ^*^(PKRs)	Below 10,000	60	22	0.1173	0.388	0.302	0.762	1.124 (0.526–2.405)	0.00124
	11,000–20,000	46	15						
	21,000–30,000	65	18	−0.1634	0.399	−0.409	0.682	0.849 (0.388–1.857)	
	Above 30,000	209	72	0.0549	0.327	0.168	0.867	1.056 (0.556–2.006)	

* P < 0.05; ^*^SE, standard error; CI, confidence interval; PKRs, Pakistani rupees.

## 4. Discussion

Pakistan is an agricultural country and its livestock is the backbone of the country. Dairy farming and livestock handling on a small scale are associated with having agricultural land. Workers on these small farms have close contact with animals, and since proper health and hygiene principles are not strictly followed, the inhabitants of these areas are at high risk for acquiring parasitic diseases. SBPDs is a group of parasitic diseases that involve the presence of snails on these livestock and farming sites. The impact of SBPDs, such as schistosomiasis and fascioliasis, require urgent investigation in the public health and livestock sectors of Pakistan. There is a large number of people who consider the two diseases a serious problem for both animals and humans. However, few studies have focused on the crucial importance of snails in the complex interactions between snails and snail-borne parasites (2, 17).

### 4.1. Knowledge about schistosomiasis and fascioliasis

More than half (57.0%) of the participants in this survey were familiar with fascioliasis and 43% were aware of schistosomiasis, but only 25.0% were aware of snails as intermediate hosts of these diseases and the majority (75.0%) of participants were unaware of the snail's role as an intermediate host. In a similar study conducted in Yemen, 92.4% of the respondents were aware of schistosomiasis and 47.2% were knowledgeable about the transmission of the disease, including transmission through snail hosts (15). In an earlier study conducted in Thailand, 55.1% of the respondents had a good level of knowledge about the mode of transmission of fascioliasis (18). In another study conducted in Ethiopia, 81.0% and 53.3% of respondents believed that the transmission of fascioliasis was caused by consumption of raw vegetables and raw meat, respectively. Approximately 24.0% believed that the disease could be transmitted to humans by a bat, but none of them were aware of the role played by snails in the transmission of fascioliasis (19).

In the life cycle of an SBPD (*Schistosoma* and *Fasciola* species) snails serve as the only intermediate host and become infected by penetrating miracidium, the larval stage of parasites and asexually replicating inside snails. As a result, thousands of cercariae are shed into the water that infects the animals who come into contact with this contaminated water (20).

### 4.2. Attitudes toward schistosomiasis and fascioliasis

During the investigation, participants were expected to protect themselves against diseases if they had sufficient knowledge and understanding of them (schistosomiasis and fascioliasis), their intermediate hosts (snails) and risks of infection. Data on participants' attitude toward snails and snail-borne diseases were collected from 507 respondents. Only 34.0% of participants attended training or an awareness session or workshop related to livestock diseases, but 74.4% (377/507) thought that SBPDs, such as fascioliasis or schistosomiasis affected the production of livestock, and 69.6% claimed to support any initiative taken to control snails that hosted *Schistosoma* and *Fasciola* spp. A comparable study from a schistosomiasis hotspot in the Philippines found that 67.1% (219/147) of the participants described schistosomiasis as a serious disease, less than half of the respondents (40.2%) did not believe that schistosomiasis was possible to prevent, and more than 80% (*n* = 187) of the respondents were willing to participate in any mass initiative taken to control and treat the disease (21).

### 4.3. Practices associated with schistosomiasis and fascioliasis

More than half of the participants claimed to use raw dairy products, such as butter, buttermilk and even raw milk without boiling it. People usually continue to follow these risky practices despite knowing their disadvantages because they consider it part of culture or tradition to remain close to nature. They are not aware of the dangers these unhygienic practices pose to the health. In a similar study regarding fascioliasis, which was conducted in Vietnam, 28.2–33.8% of the respondents used raw vegetables (22). In another study conducted in South Vietnam's coastal region, participants maintained risky practices (i.e., eating raw fish). Some of them (35.9%) considered raw fish dishes delicious, some (18.6%) stated it was part of their culture and some (10.6%) considered them a tonic (23). In response to the question about measures that were taken or would be taken to treat an animal infected with schistosomiasis or fascioliasis, 46.0% of the participants would seek professional help from an animal/veterinary care facility. Coincidently these results are quite similar to those of a 2018 study from Multan in which only 43.3% of the respondents visited qualified veterinarians for check-ups of their animals when there were infested with tick-borne diseases (24).

### 4.4. Relationship between the prevalence of schistosomiasis and fascioliasis in study area and knowledge, attitudes and practices related these diseases

The study was conducted in rural and urban areas of Punjab, Islamabad and Azad Jammu and Kashmir. Data was collected from a randomized population, which included both literate and illiterate people from different ethnic groups (i.e., Punjabi, Pakhtoon, Saraiki, and Urdu speaking). The 27.2% participants were Punjabis and the previous studies have reported the 15.2% and 13.6% prevalence of schistosomiasis during the years 2011 and 2013, respectively (9, 25) whereas a latest study was conducted in 2021 and 18 ruminants (cattle, buffalo, sheep, and goat) were screened and all were positive for fascioliasis in Punjab (26). 21.5% participants were from capital territory Islamabad and the prevalence of fascioliasis in Islamabad is documented 1.5% according to a latest analysis (27). 30% participants of the study were Kashmiri, belonged to Azad Jammu and Kashmir and a study conducted in AJK reported 40% prevalence of fascioliasis in buffaloes (28).

All these figures show the on and off prevalence of both disease and its relationship with the knowledge, attitude and practices of participants regarding these diseases. Participants had good knowledge related to fascioliasis and schistosomiasis because these diseases were prevalent in those areas at certain times.

### 4.5. Disease management for schistosomiasis and fascioliasis

A correlation between the prevalence of snails and the distribution of associated parasitic diseases has been reported (1). Several practices should be used to remove and control snails as many risks for contracting snail-borne infections during interactions with livestock were identified during the survey and the animal grazing and drinking areas were a major source of infection. More than half of the participants mentioned that the close association of family members with animals exacerbated this factor. Poor health facilities and lack of veterinary hospitals in rural areas contributed to the spread of schistosomiasis and fascioliasis. Pakistan has a large population of sheep, goats, cattle and buffaloes in rural communities and dairy farms. Remote sensing and geographic information systems techniques have been used to map the distribution of snails. These techniques not only provide information about snail habitats and dispersal areas but also predict snail-infested regions (29). After monitoring a hotspot, snail control is much easier.

Physical control measures are needed to reduce snail populations through environmental management by monitoring and eliminating snails from animals care sites and facilities, and cleaning feeding and drinking troughs, animal slaughtering areas, and butchery and animal birthing sites. Chemical control generally involves the use of a synthetic or natural chemical molluscicide, and the use of a chemical molluscicide is one of the most efficient methods of snail control (30). Although the prospective biological control of freshwater snails has gained attention because of its benefits for nature and humans when it is successful, it could have negative effects on the health of humans when it is not managed properly (31). This method of breaking the cycle of disease transmission by controlling host snail populations is a substitute for reducing the spread of such diseases due to multiple drug resistance to currently available anthelmintic drugs and the clinical unavailability of effective vaccines for SBPDs (32).

## 5. Conclusion

Similar to other neglected zoonosis, the respondents in the present study were less aware of threats related to snails, their life cycle, and preventive measures and the control of snails and SBPDs, a very few knew about the importance of controlling snails but did not know of any specific practices. Livestock is mainly reared in extensive pastures in rural areas to fulfill the meat requirements of urban communities other factors such as the unhygienic practices while rearing of domesticated livestock and common practice of home slaughtering can increase the infection rate. Livestock owners should improve their knowledge of snail-borne diseases so they can prevent and help control their spread. To make them aware of maintaining proper hygiene environment for their livestock periodic training sessions should be conducted. To create awareness for zoonotic diseases and maintaining proper hygiene environment at homes and to improve practices of people, seminars should be conducted and advertisements on media channels must be initiated locally. In urban areas, slaughtering usually occurs in meat shops by butchers who need to be aware of diseases that can spread to them while slaughtering animals. Hence, the public needs more information about SBPDs, and the control of SBPDs.

In Pakistan, there is lack of maintaining proper hygiene environment in slaughterhouses even absence of veterinary medicine, so government should take actions regarding this issue. Establishing a proper surveillance system to control the snail and snail-borne disease is much needed. A thorough surveillance should be implemented to control the snail population and the spread of SBPDs. This study provides the baseline information about lifecycle and spread of these diseases that will further stimulate interest in future research in Pakistan.

## Data availability statement

The original contributions presented in the study are included in the article/supplementary material, further inquiries can be directed to the corresponding authors.

## Ethics statement

The studies involving human participants were reviewed and approved by Institutional Review Board of Comsats University Islamabad. The patients/participants provided their written informed consent to participate in this study.

## Author contributions

SR collected the data and authored the paper following discussions with HA and JC. HA designed the study. SR and SK applied the study's methodology. HA, MA, SS, FC, NB, SKN, SW, JZ, and JC revised the paper. All authors have read and agreed to the published version of the manuscript.
